# Innovative Hybrid Approach for Masked Face Recognition Using Pretrained Mask Detection and Segmentation, Robust PCA, and KNN Classifier

**DOI:** 10.3390/s23156727

**Published:** 2023-07-27

**Authors:** Mohammed Eman, Tarek M. Mahmoud, Mostafa M. Ibrahim, Tarek Abd El-Hafeez

**Affiliations:** 1 Computer Science Department, Faculty of Computing and Artificial Intelligence, Beni Suef University, Beni-Suef 62511, Egypt; 2 Computer Science Department, Faculty of Science, Minia University, Minia 61519, Egypt; 3 Computer Science Department, Faculty of Computers and Artificial Intelligence, University of Sadat City, Sadat City 32897, Egypt; tarek@fcai.usc.edu.eg; 4 Electrical Engineering Department, Faculty of Engineering, Minia University, Minia 61519, Egypt; mostafa.ibrahim@mu.edu.eg; 5 Computer Science Unit, Deraya University, Minia 61765, Egypt

**Keywords:** face masks problem, robust principal component analysis, particle swarm optimization, KNN

## Abstract

Face masks are widely used in various industries and jobs, such as healthcare, food service, construction, manufacturing, retail, hospitality, transportation, education, and public safety. Masked face recognition is essential to accurately identify and authenticate individuals wearing masks. Masked face recognition has emerged as a vital technology to address this problem and enable accurate identification and authentication in masked scenarios. In this paper, we propose a novel method that utilizes a combination of deep-learning-based mask detection, landmark and oval face detection, and robust principal component analysis (RPCA) for masked face recognition. Specifically, we use pretrained ssd-MobileNetV2 for detecting the presence and location of masks on a face and employ landmark and oval face detection to identify key facial features. The proposed method also utilizes RPCA to separate occluded and non-occluded components of an image, making it more reliable in identifying faces with masks. To optimize the performance of our proposed method, we use particle swarm optimization (PSO) to optimize both the KNN features and the number of k for KNN. Experimental results demonstrate that our proposed method outperforms existing methods in terms of accuracy and robustness to occlusion. Our proposed method achieves a recognition rate of 97%, which is significantly higher than the state-of-the-art methods. Our proposed method represents a significant improvement over existing methods for masked face recognition, providing high accuracy and robustness to occlusion.

## 1. Introduction

The COVID-19 pandemic has affected how people interact with each other. To mitigate the spread of the pandemic, governments have instituted rules such as wearing masks, staying away from others, and staying at home. Wearing masks helps protect people from the epidemic; however, it makes it difficult for facial recognition systems to recognize people’s faces.

It is hard to keep important information safe using traditional passwords and security measures; therefore, researchers have been focusing on a special kind of technology called biometric technology that is proficient at maintaining security and is very difficult for people to imitate [1]. Consequently, face biometric technology has received more attention to recognize a person correctly or recognize facial emotions [2,3]. Biometric facial recognition technology is widely used in various applications such as security systems, access control, and law enforcement.

With the widespread use of masks due to the COVID-19 pandemic, facial recognition systems face significant challenges in identifying masked faces. This has created a need for new methods that can accurately identify individuals wearing masks. Because of the COVID-19 pandemic, the masked face detection and recognition problems have become the most important area to innovate new methods and algorithms to detect and recognize people who are wearing or not wearing mask to reduce and prevent spread of COVID-19. Matching a masked face with unmasked or masked faces is the goal of the masked face recognition problem (MFR).

### 1.1. Motivation

Face masks are used in a variety of settings and industries, and there are many jobs that require the use of face masks to protect workers and the public. Some examples of jobs that commonly require the use of face masks include *healthcare workers, food service workers, construction workers, manufacturing workers, retail workers, hospitality workers, transportation workers, educational workers, and public safety workers*.

**Healthcare workers,** including doctors, nurses, and other medical professionals, use face masks to protect themselves and their patients from the spread of infectious diseases. 

Workers in the **food service industry**, including cooks, servers, and other staff, use face masks to prevent the spread of germs and bacteria in food preparation and service areas. This helps to protect both workers and customers from the spread of infectious diseases.

**Construction workers** often work near one another, making it difficult to maintain social distancing. Face masks are used to protect workers from the spread of respiratory droplets on job sites and in work trailers. 

**Workers in manufacturing** settings often work in close proximity to one another on assembly lines or in production areas. Face masks are used to protect workers from the spread of respiratory droplets and other airborne particles. 

**Retail workers**, including cashiers and sales associates, use face masks to protect themselves and customers from the spread of infectious diseases. This is particularly important in situations where social distancing is not possible, such as in crowded stores or during busy shopping periods. 

For **hospitality workers**, workers in the hospitality industry, including hotel staff, restaurant servers, and bartenders, use face masks to protect **themselves** and patrons from the spread of infectious diseases. This is particularly important in situations where social distancing is not possible, such as in dining areas or at bars. 

**Transportation workers**, including bus drivers, taxi drivers, and airline staff, use face masks to protect themselves and passengers from the spread of infectious diseases. This is especially important in situations where social distancing is not possible, such as in crowded buses or airplanes. 

**Educational workers**, including teachers, school staff, and other workers in educational settings, use face masks to protect themselves and students from the spread of infectious diseases. This is particularly important in situations where social distancing is not possible, such as in classrooms or during school events. 

**Public safety workers**, including police officers, firefighters, and other public safety workers, use face masks to protect themselves and the public from the spread of infectious diseases. This is particularly important in situations where close contact with others is likely, such as during emergency responses or public events.

### 1.2. Contribution

Face detection systems face a number of challenges when it comes to detecting faces that are partially or fully covered by face masks. Here are some of the key challenges that face detection systems encounter when faced with masked faces:

- **Reduced accuracy**: Face detection systems use machine learning algorithms to identify and track faces. When a face is partially or fully covered by a mask, the system may not be able to accurately identify the face, leading to reduced accuracy in the detection process.
- **False positives and negatives**: In some cases, face detection systems may mistake a mask for a face or may fail to detect a face that is partially covered by a mask. This can lead to false positives or negatives, which can compromise the accuracy of the system.
- **Need for retraining**: Face detection systems that were trained on unmasked faces may not perform well when faced with masked faces. To accurately detect faces that are partially or fully covered by masks, the system may need to be retrained using data that includes masked faces. This can be time-consuming and resource intensive.
- **Dependence on other features**: In some cases, face detection systems may rely on other features, such as the shape of the head or the position of the eyes, to accurately identify faces. When a face is partially or fully covered by a mask, these other features may not be visible, making it more difficult for the system to accurately identify the face.
- **Privacy concerns**: The use of face detection systems, particularly in public spaces, can raise privacy concerns. When individuals are wearing masks, the system may not be able to accurately identify them, which can lead to **concerns** about government surveillance and tracking.
- **Limitations in low-light conditions**: Face detection systems may also be limited in low-light conditions, particularly when individuals are wearing masks. This can make it difficult for the system to accurately detect and track faces, leading to reduced performance and increased false positives.
- **Adaptation to different mask types**: There are many different types of masks available, including surgical masks, cloth masks, and N95 masks. Each type of mask may present different challenges for face detection systems, which may need to be adapted to detect faces that are partially or fully covered by different types of masks.
- **Impact on system performance**: When a face detection system is faced with many masked faces, the performance of the system may be impacted. This can lead to slower processing times, increased false positives, and reduced accuracy.

To address this issue, an integrated approach is proposed, combining the following: (1) Two pretrained deep-learning-based algorithms; one is based on Single Shot Multibox Detector (SSD)-MobileNetV2 for mask detection and the other is based on MediaPipe model for landmark and oval face detection; (2) Robust Principal Component Analysis (RPCA) for robust occlusion and accuracy in identifying faces with masks face recognition; (3) the K-Nearest Neighbors (KNN) classifier for face recognition; (4) the Particle Swarm Optimization (PSO) algorithm to select the features used for training the KNN classifier and optimize the k number.

### 1.3. Paper Organization

The paper is organized as follows: In Section 2, the literature of existing studies is presented. Section 3 introduces our proposed hybrid method in detail. Section 4 presents the experimental results and discussion. Section 5 concludes this paper. Finally, Section 6 points out the future research ideas.

## 2. Related Work

The literature on masked face recognition is reviewed in this section. Previous approaches can be divided into two categories: traditional machine learning approaches and deep learning approaches. In recent years after the pandemic, many researchers devoted their efforts to finding a solution to the problem of mask confusion and its impact on the face recognition task [4,5]. In this study [6], Neto et al. aim to evaluate the different approaches followed for both Masked Face Recognition (MFR) and Occluded Face Recognition (OFR), find linked details about the two conceptually similar research directions, and understand future directions for both topics. The analysis presented sustains the interoperable deployability of MFR methods on OFR data sets when the occlusions are of a reasonable size. Thus, solutions proposed for MFR can be effectively deployed for general OFR.

For unmasked face recognition tasks, many researchers proposed methods based on machine learning methods such as KNN, However, these classical methods [7] are sensitive and affected by occlusion caused by masks. On the other hand, some researchers proposed a consistent sub-decision network (CSDN) that specifically targets low-quality masked face images [8]. In this work, the authors proposed a consistent sub-decision network that achieves more consistent model inferences. This method makes the network concentrate more on the upper faces without occlusion and extracts more discriminative features.

For masked face detection task only [9], a CNN-based bi-directional and directional long-short term memory network is proposed for determining whether a person is wearing a face mask or not.

Recently, most researchers have proposed methods that use a combination of deep-learning-based mask detection and face recognition. Convolutional neural network (CNN) models have been used to develop a number of face detection approaches specifically for face mask identification that use the precise findings of deep learning algorithms in the problem of masked face recognition [10,11,12]. Aswal and Tupe et al. [13] proposed a single camera masked face detection and identification method based on two approaches, where they considered a single-step pretrained YOLO-face/trained YOLOv3 model on a set of known individuals and a two-step method based on a pre-trained one-stage feature pyramid detector network RetinaFace. 

By utilizing a mobile application named MadFaRe [14], the authors suggested and verified a technique to discriminate between masked, unmasked, and incorrectly masked persons. Eight object detection models and four face detection models make up the Adhikarla and Davison [10] framework. To enhance face mask identification, many models are being used. They use “with-mask”, “without-mask”, and “unsure” to describe themselves. Despite the results of the accuracy improvement, there are still costs associated with time complexity and computation.

In ref. [15], a novel facial recognition technique for partial occlusion was presented, employing Multi-Task Cascaded Neural Network (MTCNN) for face detection, and removing the LBP (Local Binary Pattern) features from the non-occlusion area, which is a feature that effectively depicts the texture of face images and can further enhance the robustness of face recognition methods. Ding et al. [11] introduce the two-branch CNN, which consists of a global branch for discriminative global feature learning and a partial branch for latent component identification and discriminative partial feature learning. 

The top convolutional feature maps were created in the global branch using the ResNet-50 model. While utilizing the latent part detection approach, the most discriminative latent area in masked facial images has been localized in the local section. 

By integrating CNN parameters in the two branches, the two branches can be made smaller and more useful features can be retrieved, which is the new information the author supplied. This will increase the functioning of masked face systems. Two pre-trained CNN models called YOLOv3 and faster regions with convolutional neural networks (R-CNN) were used by Singh et al. [16] to complete and improve the results of this challenge. The performance and accuracy of masked face detection are improved by these integrated models, although complex problems remain. In the same manner as [16], two CNN models were utilized by Zhu et al. [17]. The first level uses the Dilation RetinaNet face location (DRFL) network to find faces in crowds, while the second level uses the SRNet20 network to classify faces using masks.

Many papers introduced the transfer learning mechanism using different deep learning models. An InceptionV3 pre-trained model is used to adopt the transfer learning approach, and the simulated masked face data set (SMFD) is used [18]. Using the transfer learning, another system made up of three different models for the detection phase, including the support vector machine (SVM), decision trees, and ensemble algorithm embedded with ResNet50, is then introduced, using the ResNet50 deep learning model for the feature extraction phase. The RMFD, SMFD, and LFW data sets are used to test the system [19].

A system developed by Suresh et al. [20] using MobileNet was demonstrated as being able to recognize individuals who are not wearing masks and transmit the image to permitted staff members. In a separate work by Lodh et al. [21], a model was created by fine-tuning MobileNetv2 on a collection of masked and unmasked images in the suggested system. At the program’s end, a precision of over 98% was achieved. This technique finds those who are not hiding their faces before identifying their faces.

In a separate study, the MTCNN [22] is used to identify the face in masked face photographs and train the system using the LeNet algorithm to compare the accuracy of the masked and unmasked classifications. The deep learning methods are promising methods for MFR. However, the necessity for large-scale masked face data sets for training is shown by the promising candidate deep learning and such data sets are not easily accessible or sufficient. 

Saleh et al. [23] proposed a two-stage deployment of the concept. The texture and color moments feature from the facial photographs are extracted in the first phase using 3705 images by hybridizing these qualities. In the following step, the face images are classified using Multi-Layer Perceptron (MLP) and the extracted characteristics. Wu [24] developed a system based on subsampling and provided a novel method for the recognition process. An attention machine neural network and a ResNet were used to put the technique into practice. Real world masked face recognition data set (RMFRD) and synthetic masked face recognition data set (SMFRD) databases were used in the studies, and the findings show strong performance due to the cheap time cost and high accuracy rate.

Ullah et al. [25] propose a novel DeepMaskNet framework capable of both face mask detection and masked facial recognition. The authors also developed a large-scale and diverse unified mask detection and masked facial recognition (MDMFR) data set to measure the performance of both the face mask detection and masked facial recognition methods. In ref. [26], an efficient and effective model is proposed for real-time face mask detection with the potential to be deployable over edge devices. Their proposed model is based on MobileNetV2 architecture that extracts salient features from the input data that are then passed to an autoencoder to form more abstract representations prior to the classification layer.

For face mask detection, Farman et al. in [27] focus on real-time face mask detection in developing countries, addressing the challenges of implementing mask-wearing guidelines. In ref. [28], the recognition of masked faces is discussed and the authors highlight the lack of improvement in face recognition accuracy despite extensive natural exposure. The Spartan face mask detection and facial recognition system is introduced in [29], which combines face mask detection and facial recognition technologies. Kwak et al. [30] explore masked face detection and recognition using transfer learning techniques. In the case of multiple scenes [31], a new model is proposed for masked face recognition.

Moreover, a convolutional visual self-attention network [32] is utilized as a masked face recognition method. The authors propose an attention mechanism that focuses on the visible regions of the face while considering the occluded areas due to masks. This approach addresses the challenges posed by face masks and achieves improved recognition accuracy. In ref. [33], deep metric learning and the FaceMaskNet-21 architecture are employed for masked face recognition. They leverage the power of deep learning to learn discriminative features from masked faces and utilize a metric learning framework for enhanced recognition performance. This work highlights the effectiveness of deep learning models in handling mask occlusions. Yuan et al. [34] propose a so-called MSML framework, which enhances occlusion-robustness in face recognition through multi-scale segmentation-based mask learning. The authors leverage segmentation techniques to generate masks that separate visible face regions from occluded regions caused by masks. This approach improves the robustness of face recognition algorithms against mask occlusions. In ref. [35], the authors propose the PLFace method, which incorporates progressive learning for face recognition with mask bias. The method progressively adapts the face recognition model to masked face data, gradually reducing the bias introduced by masks. This approach improves recognition accuracy for both masked and unmasked faces. 

The research of Fang et al. [36] focuses on the challenges posed by real masks and spoof faces in masked face presentation attack detection. The authors investigate the vulnerability of face recognition systems to attacks using real masks and spoofed faces. They discuss the importance of developing robust detection methods to prevent unauthorized access and ensure the integrity of face recognition systems. Shatnawi et al. [37] propose a deep learning approach for masked face identification where they use deep convolution neural network (DCNN)- and MobileNetV2-based transfer learning models to detect face masks in public places to curtail the spread of Coronavirus. The models are trained, validated, and tested on different data sets, achieving high accuracy. Using an attention-based mechanism, Pann et al. [38] propose a new method for masked face recognition by integrating a cropping-based approach with the Convolutional Block Attention Module (CBAM). The optimal cropping is explored for each case, while the CBAM module is adopted to focus on the regions around eyes.

Combining between the classical machine learning and the subfield deep learning, Oumina et al. [39] classify the retrieved features under assessment using classifiers like SVM and KNN in conjunction with pre-trained deep learning models like VGG19, Xception, and MobileNetV2. 

Our proposed hybrid method follows the category of combining classical machine learning such as KNN and CNN. Our proposed method addresses the limitations of using the deep learning method alone where a large data set is required. To that end, our proposed method consists of a combination of pretrained mask detection, landmark detection based on pretrained deep learning, and robust principal component analysis for feature extraction and KNN classifier for face recognition. Table 1 presents a Comparison of Different Methods for Face Mask Recognition and Detection

## 3. Methodology

This section presents the methodology employed in this paper, which proposes a novel approach to masked face recognition.

The proposed method integrates deep-learning-based mask detection, landmark and oval face detection, and robust principal component analysis (RPCA) to achieve accurate recognition of masked faces. The following steps outline the methodology:

- **Mask Detection**: A pretrained ssd-MobileNetV2 model is utilized to detect the presence and location of masks on a face. This deep-learning-based approach effectively identifies whether a person is wearing a mask or not.
- **Landmark and Oval Face Detection**: Landmark and oval face detection techniques are employed to identify key facial features. This step helps in precisely localizing facial landmarks and obtaining a better representation of the face.
- **Robust Principal Component Analysis (RPCA)**: RPCA is utilized to separate occluded and non-occluded components of an image. By extracting the non-occluded components, the proposed **method** becomes more reliable in identifying faces with masks, as it focuses on the visible facial features.
- **Optimization using Particle Swarm Optimization (PSO)**: To enhance the performance of the proposed method, particle swarm optimization (PSO) is employed. PSO optimizes both the KNN features and the number of k (nearest neighbors) for KNN, resulting in improved accuracy and robustness.

Pseudocode of our proposed method are mentioned below:
**Step 1:** Input Masked Face Image. **Step 2:** Run Pretrained Mask Detection and Landmark Detection.    **2.1:** Mask Segmentation and Conversion to Black Pixels. **Step 3:** Prepare, input data set and set the parameter. **Step 4:** Normalize the Data. **Step 5:** Initialize arrays for feature vectors and labels. **Step 6:** Use Robust PCA for dimension reduction. **Step 7:** Extract features using LBP.    **7.1:** Convert binary feature selection vector to logical indexing vector. **Step 8:** Split the data into training and testing sets. **Step 9:** Use PSO in feature selection.    **9.1:** Define the fitness function for PSO-KNN. **Step 10:** Use PSO in optimizing the number of k. **Step 11:** Use PSO-KNN for face recognition with optimized k value. **Step 12:** Evaluate the performance of the PSO-KNN classifier.    **12.1:** Train KNN classifier using training data and optimized k.    **12.2:** Test the classifier.

**Figure 1:**
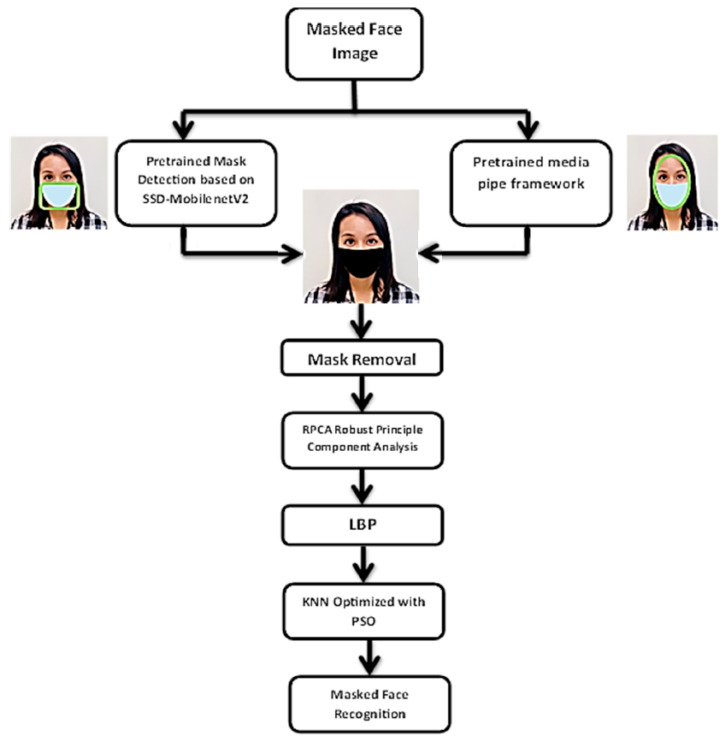
The Flowchart of the Proposal Method.

**Figure 2:**
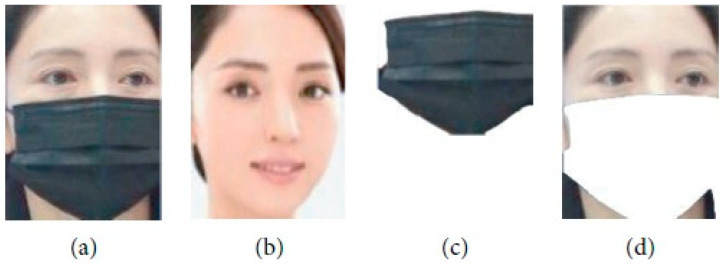
Example of mask separation. (**a**) Mask face image. (**b**) Reference image. (**c**) Separated mask. (**d**) Separated face.

We will explain every step of our proposed methods in the following subsections in detail. Figure 1 shows our proposed pipeline and Figure 2. Shows an example of mask separation, mask face image, reference image, separated mask, and separated face. 

The distance between a camera and an object can be calculated using various methods, depending on the type of camera, the environment, and the object being measured. Here are some of the most common methods:

- Triangulation: This method involves using two cameras to measure the distance to an object. The cameras are placed at a known distance from each other and are pointed at the object. By comparing the position of the object in the two camera images, the distance can be calculated using triangulation.
- Time-of-flight: This method involves emitting a signal, such as a laser or infrared light, from the camera towards the object and measuring the time it takes for the signal to bounce back. The distance can be calculated by multiplying the time of flight by the speed of light.
- Stereo vision: This method uses two cameras placed a known distance apart to create a 3D image of the scene. By comparing the difference in position of an object in the two camera images, the distance to the object can be calculated.
- Structured light: This method involves projecting a pattern of light onto the object and measuring the distortion of the pattern caused by the object’s surface. The distance can be calculated by analyzing the distortion of the pattern.
- Focus: This method involves adjusting the focus of the camera lens until the object is in sharp focus. The distance can be calculated by using the known focal length of the lens and the distance from the lens to the image sensor.

We used the focus technique. The accuracy of these methods can vary depending on the environment, lighting conditions, and the size and shape of the object being measured.

### 3.1. Deep-Learning-Based Mask Detection and Face Oval Detection

**Figure 3:**
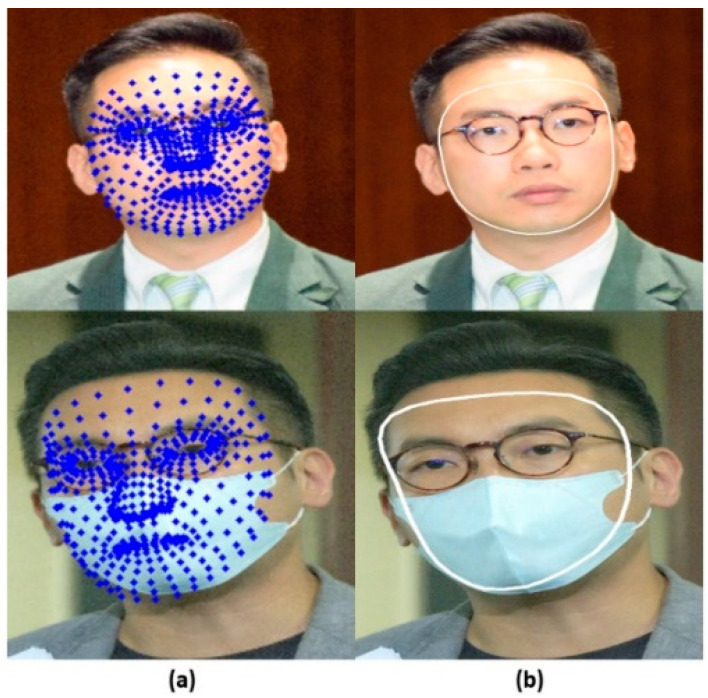
(**a**) Face Landmarks Detection and (**b**) Face Oval Detection.

The technique of recognizing distinct features on a person’s face, such as the corners of the eyes, the tip of the nose, and the borders of the lips, is known as facial landmark detection. Machine learning techniques are frequently used by facial landmark identification algorithms to recognize these landmarks. In our work, MediaPipe framework [40] is used for detecting the landmarks of the face images. By using MediaPipe framework, we can detect the face oval which is the outline of the face constructed by connecting the outer face landmarks as shown in Figure 3. We use a pretrained deep-learning-based mask detection method for detecting masks. This pretrained model is based on SSD-MobileNetV2. SSD-MobileNetV2 is a widely used object detection model that is specifically designed to operate quickly and accurately on devices with limited computational power, such as smartphones. It employs a combination of a base network called MobileNetV2 and a detection layer known as SSD (Single Shot Detector) to predict the bounding boxes and class labels of objects within an image.

The reason why SSD-MobileNetV2 is chosen for object detection tasks is due to its numerous advantages over other models, such as the following:

- It is lightweight and efficient, meaning it has fewer parameters and operations than other models, which reduces memory and power consumption. It can achieve real-time inference (30 frames per second) even on mobile devices.
- It employs depthwise separable convolutions, which are a type of convolution that divides the standard convolution into two steps: a depthwise convolution that applies a single filter to each input channel, and a pointwise convolution that combines the outputs of the depthwise convolution. This reduces the number of computations and parameters by a factor of 8 to 9.
- It utilizes inverted residual blocks, which are a type of residual block that have thin bottleneck layers at the input and output, and a thick expansion layer in the middle. This allows the network to learn more complex features with fewer parameters and computations.
- It is one-stage, meaning it directly outputs the bounding boxes and class labels without using any intermediate steps, such as region proposals or feature pyramids. This makes it faster and simpler than two-stage models, such as Faster R-CNN or Mask R-CNN.

### 3.2. Robust Principal Component Analysis

One of the well-known dimension reduction techniques is the principal component analysis (PCA) [41]. In computer vision, it is used to represent an image with a relatively small dimensional feature vector. However, PCA is fragile with respect to outliers. For tackling this drawback, Candès et al. developed a statistical method called Robust PCA (RPCA) [42], where RPCA is used for decomposing data into its principal components (i.e., the underlying structure of the data), while also identifying and removing outliers and noise. This makes RPCA a powerful tool for a variety of applications, including image and video processing, signal processing, and machine learning. It is often used in situations where the data contain noise or outliers that can distort the results of traditional PCA. In our case, the outliers are the mask pixels that occlude the lower part of the face images.

**Figure 4:**
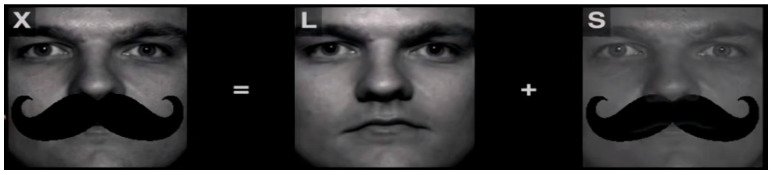
RPCA Technique.

Before using RPCA, the masks that occlude the face images are segmented by extracting the intersection region between the bounding box surrounding the masks and the face ovals detected using mediapipe library. Then, the segmented masks are converted to black pixels to not disturb the RPCA, where we investigate that the colored masks are not classified well as outliers. After that, RPCA is used for obtaining the low-rank matrix of face features. Figure 4 shows an example of the RPCA technique.

In our algorithm, RPCA assumes that the matrix consisting of face images features vectors (denoted as X) is a combination of a low-rank component which represents the eigen faces and a sparse component that contain the occlusion pixels. The RPCA method aims to factorize an input matrix X into the sum of a low-rank matrix L and a sparse matrix S such that X = L + S. This can be formulated as the following optimization problem,

(1) $$\underset{L,S}{min}\;\mathrm{rank}\;\left(L\right)+∥S{∥}_{0}\;\mathrm{subject}\;\mathrm{to}\;L+S=X,$$

where $∥\;.{∥}_{0\;}$denotes the L_0_ norm. We can find the best L and S with a high probability by using a simpler way called convex relaxation, where the rank is relaxed to nuclear norm and the L_0_ norm is relaxed to the L_1_ norm. After convex relaxation, the equation becomes as follows,

(2) $$\underset{L,S}{min}\;∥L{∥}_{*}+\lambda ∥S{∥}_{1}\;\mathrm{subject}\;\mathrm{to}\;L+S=X$$

where $∥\;.{∥}_{*\;}$denotes the nuclear norm and $∥\;.{∥}_{1\;}$denote the L_1_ norm. There are several algorithms that can be used to perform RPCA, including the Principal Component Pursuit (PCP) algorithm and the Alternating Direction Method of Multipliers (ADMM) algorithm [42]. These algorithms solve the optimization problem required for RPCA and can efficiently and effectively separate the low-rank and sparse components. After obtaining the low-rank matrix L which represents the eigen faces without holes or black pixels, KNN is then used for face recognition.

### 3.3. K-Nearest Neighborhood Algorithm and Particle Swarm Optimization (PSO) for Face Recognition

People can be recognized by looking at certain things in their faces, like the color and texture. Texture is especially important for biometric face recognition because it helps the computer recognizes patterns in the face image. Researchers use a well-known method called the local binary pattern (LBP) to extract the most important features of the face image. LBP is effective because it works even if the face image looks different under conditions such as lighting, the person’s facial expressions, or pose. Finding these important parts of the face is very important for computers to recognize people’s faces accurately.

Feature selection is an important step for a wide range of machine learning approaches and computer vision tasks, including facial recognition. Feature selection aims to identify the most relevant and informative features in the data, while eliminating the redundant and irrelevant ones that may negatively affect the classifier performance. This can help to reduce the dimensionality of the feature space, which can in turn improve the accuracy and efficiency of recognition algorithms [43]. Moreover, the KNN classifier is a facial recognition method that is both easy to use and efficient computationally. However, the initialization of the parameter k causes it to suffer. Therefore, k is also required to optimize.

To select the features used for training the KNN classifier and optimize the k number, evolutionary algorithms such as Particle Swarm Optimization (PSO) and Genetic Algorithm (GA) are used because they are global metaheuristic optimization techniques, where they are not affected by the issue of local minima. Based on the results of [7], PSO is the best choice used for optimum features selection and k selection. 

## 4. Experimental Settings and Results

### 4.1. Experimental Settings

**Figure 5:**
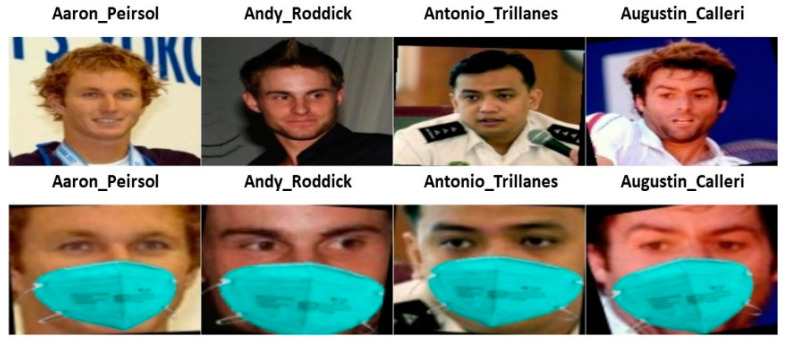
Pairs of simulated masked and non-masked face images.

This subsection mentions the setting for the experiments. Two data sets—simulated and real masked face data sets—are used for testing the performance of different masked face recognition methods. The simulated one is the Labeled Face in the Wild Stimulated Masked Face Data Set (LFW-SMFD) [44]. The data set consists of 13,117 faces of 5713 people. Sample images from simulated masked and non-masked faces images are shown in Figure 5.

**Figure 6:**
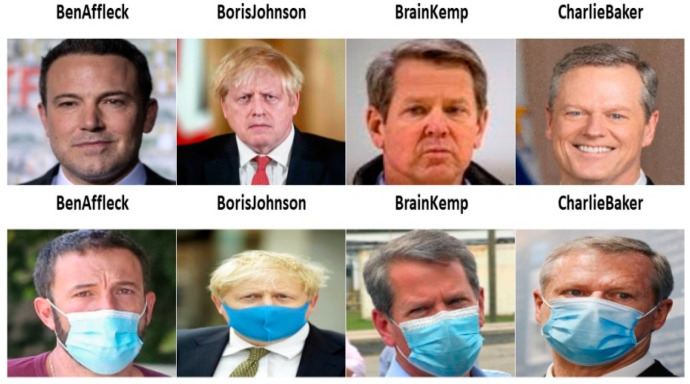
Pairs of real masked and non-masked face images.

For the real data set, we chose to use actual photographs of people wearing masks rather than computer-generated masked face images to provide a more realistic testing environment. We searched Google for masked pictures of well-known people, such as politicians and celebrities, to build our database. The photographs we chose were carefully chosen to be of the highest caliber and to be free of duplication. Sample images from real masked and non-masked faces images are shown in Figure 6.

### 4.2. Experimental Results and Discussion

In this section, the performance of our proposed algorithm will be evaluated compared by other methods such as the method of Ejaz et al. [45] which uses the PCA technique, Latent Part Detection method [11], and Rodriguez et al.’s [46] method that uses the mixture of Gaussian method.

The experimental results were obtained using a computer system running Windows 11 Home as its operating system. The processor used in this system is the AMD Ryzen™ 7 6800H, which has a maximum boosted frequency of up to 4.7 GHz, 3 MB L16 cache, 8 cores, and 16 series processing. The system utilizes AMD’s on-chip system technology for its chipset. The graphics cards are separate and consist of an NVIDIA^®^ GeForce RTX™ 3070 Ti Laptop GPU with 6 GB of dedicated GDDR8 memory. The system is equipped with 16 GB of DDR4800-16 MHz RAM (2 × 8 GB) with transfer rates of up to 4800 MB/s. Additionally, the system has a Gen4 SSD with 1 TB of storage. For video conferencing and recording, the system includes an HP Wide Vision 720p HD camera with temporary noise reduction and integrated dual-array digital microphones.

**Figure 7:**
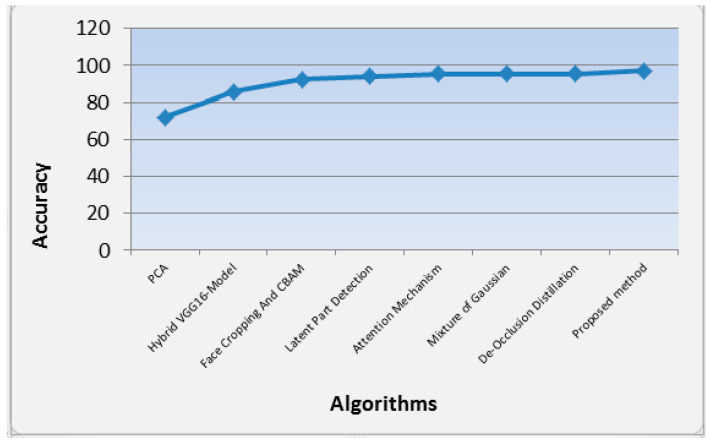
Analysis of the performance of the accuracy of different algorithms in masked face recognition.

Table 2 and Figure 7 shows the performance of our proposed methods compared to the other methods in terms of accuracy in recognition of masked images. From Table 1, it is clearly shown that our proposed method achieves accuracy 97% for the masked data set, which outperforms the compared methods. Moreover, it is evident that the performance of PCA alone [45] is the worst one compared with the other results.

**Figure 8:**
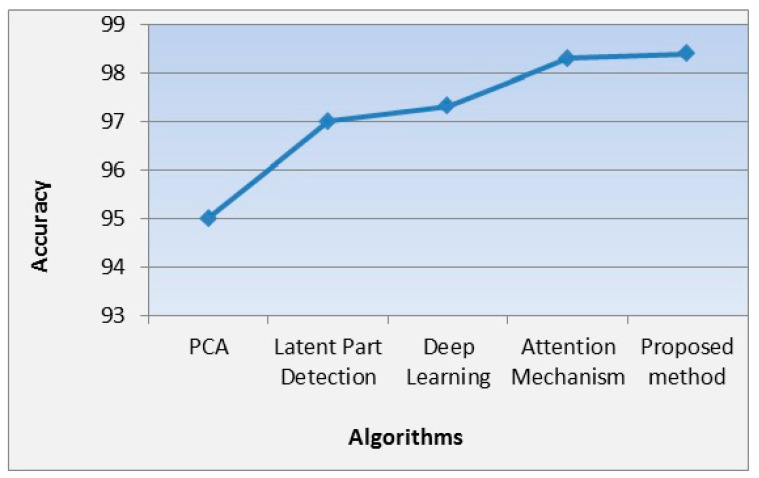
Analysis of the performance of the accuracy of different algorithms in unmasked face recognition.

Table 3 and Figure 8 show the performance of our proposed methods compared to the other methods in terms of accuracy in recognition of unmasked images. From Table 2, it is clearly shown that our proposed method achieves accuracy 98.4% for the unmasked data set, which outperforms the compared methods. Moreover, it is evident that the performance of PCA alone [25] is also the worst one compared with the other results.

For the KNN part, the features are obtained from LBP after applying the RPCA then the KNN classifier optimized by PSO is used and it is denoted as PSO-KNN. The parameters PSO algorithm is reported in Table 4. The effectiveness of the PSO-KNN algorithm has been evaluated in comparison to conventional benchmark classifiers. 

Table 5 describes the accuracy of the proposed KNN optimized by PSO compared with unoptimized k number of KNN in the cases of using different optimization techniques such as PSO and GA for feature extraction.

The results show that PSO-KNN outperforms KNN in all scenarios, achieving a higher classification accuracy. For example, when using actual features, KNN achieved an accuracy of 89%, while PSO-KNN achieved an accuracy of 93%. When using GA for feature selection, KNN achieved an accuracy of 94%, while PSO-KNN achieved an accuracy of 96%. When using PSO for feature selection, KNN achieved an accuracy of 95%, while PSO-KNN achieved an accuracy of 98%, which is the highest accuracy among all methods.

The results demonstrate that PSO-KNN is a more effective algorithm for masked face recognition, achieving higher accuracy compared to KNN, especially when PSO is used for feature selection and optimizing the value of k.

### 4.3. Execution Time Results

The SSD-Mobilenet is a real-time object detection model that is designed to run at a high frame rate of 46 frames per second. This means that it takes approximately 0.02 s to process each frame and detect objects within it. The model achieves this high performance by combining the Single Shot Detector (SSD) algorithm with the efficient MobileNet architecture.

In contrast, the RPCA and KNA algorithms have a lower frame rate of 4 frames per second and are not considered to be real-time. These algorithms take approximately 0.3 s to process each frame, resulting in a total processing time of 0.32 s. While these algorithms may be effective for certain tasks, they are not suitable for applications that require real-time object detection.

The SSD-Mobilenet model is a highly efficient and effective real-time object detection solution that can process frames at a much faster rate than other algorithms such as RPCA and KNA. Its ability to accurately detect objects in real-time makes it a valuable tool for a wide range of applications, from self-driving cars to security systems and beyond.

## 5. Discussion

The proposed method of combining mask detection, facial landmark detection, oval face detection, and RPCA represents a promising approach for achieving higher accuracy in masked face recognition. By detecting and localizing the mask and key facial features, the method is able to extract relevant non-occluded facial components for recognition. The use of RPCA to separate occluded and non-occluded components helps improve occlusion robustness. Finally, optimizing the KNN features and parameters using PSO further enhances performance. This is evidenced by the achieved recognition rate of 97%, outperforming existing methods. The proposed method thus demonstrates significant advancements for addressing the challenges of masked face recognition. We can summarize our contribution discussion as follows:

- The combination of deep learning and traditional computer vision techniques achieves state-of-the-art performance.
- The mask detection and facial feature localization improve robustness to occlusion.
- The use of RPCA to separate occluded and non-occluded features is effective for masked face recognition.
- Optimizing the KNN parameters using PSO further boosts the accuracy.
- The 97% recognition rate significantly outperforms existing methods.
- The proposed method represents an important advancement for masked face recognition.
- The approach is potentially applicable to real-world scenarios where face masks are common.
- The methodology can be extended to other types of facial occlusion.
- The techniques are generalizable and could be applied to other computer vision tasks.
- The approach provides a foundation for future research on masked face recognition.

## 6. Limitations

While the proposed method shows high accuracy and robustness, there are several limitations that could be addressed in future work.

- The method relies on a data set of individuals wearing different types of masks. The performance may degrade for novel mask types not present in the data set. Collecting a more comprehensive masked face data set could help address this.
- The accuracy may be sensitive to mask placement, facial poses, and lighting conditions. Collecting a more diverse data set that covers more mask variations, poses, and conditions could help improve robustness.
- The method has only been evaluated on still images and may not generalize well to video. Extending the method to video-based masked face recognition could enable real-world applications.
- The computational cost of combining multiple deep learning and computer vision techniques may be high. Methods for optimizing efficiency could be explored to enable on-device applications.
- The ability to handle occlusion from other objects has not been evaluated.

## 7. Future work

In future work, we can focus on extending our proposed method to handle other types of occlusions, such as sunglasses, scarfs, and hats, which can also pose challenges to face recognition systems. Additionally, integrating other biometric modalities, such as voice and fingerprint, can further enhance the accuracy and reliability of the system. Another direction for future work is to investigate the privacy implications of masked face recognition. As masks have become a ubiquitous part of daily life, concerns about facial recognition technology’s impact on privacy have increased. 

Our proposed method relies on face recognition, which raises concerns about the potential for misuse and abuse of the technology. Therefore, future research can focus on developing ethical guidelines and regulations for the use of masked face recognition technology.

## 8. Conclusions

The use of face masks has become essential in various industries and jobs, necessitating the development of effective masked face recognition technologies. In this paper, we proposed a novel method that combines deep-learning-based mask detection, landmark and oval face detection, and robust principal component analysis (RPCA) for accurate masked face recognition. Our proposed method utilizes pretrained ssd-MobileNetV2 for mask detection and RPCA to separate occluded and non-occluded components of an image. To optimize the performance of our proposed method, we used particle swarm optimization (PSO) to optimize both the KNN features and the number of k for KNN. Experimental results showed that our proposed method outperformed existing methods in terms of accuracy and robustness to occlusion, achieving a recognition rate of 97%, significantly higher than the state-of-the-art methods. Overall, our proposed method represents a significant improvement over existing methods for masked face recognition, providing high accuracy and robustness to occlusion.

## Figures and Tables

**Table 1:** Comparison of Different Methods for Face Mask Recognition and Detection.

Study	Method	Drawbacks	Advantages
Optimization of K-nearest neighbor using particle swarm optimization for face recognition	KNN classifier with PSO feature selection	- High computational cost—Sensitive to noise and outliers	- Simple and easy to implement—Robust to variations in pose, expression, and illumination
Consistent Sub-Decision Network for Low-Quality Masked Face Recognition	CNN with sub-decision network and consistency loss	- Requires large-scale training data—May not generalize well to unseen masks	- Improves the quality of masked face features—Enhances the consistency between masked and unmasked faces
Face mask detection on real-world Webcam images	CNN with transfer learning from ImageNet	- Limited by the quality of webcam images—May not detect masks with complex patterns or colors	- Fast and accurate on webcam images—Can be easily deployed on web applications
Masked face recognition with latent part detection	CNN with latent part detection module	- Depends on the accuracy of part detection—May fail on heavily occluded faces	- Learns discriminative features for each facial part—Adapts to different types of masks
Masked face recognition with convolutional neural networks and local binary patterns	CNN with LBP feature extraction	- Requires manual alignment of face images—LBP features may lose some information	- Combines global and local features—Robust to illumination changes
Single camera masked face identification	CNN with Siamese network and triplet loss	- Needs a large number of triplets for training—May suffer from hard negative mining problem	- Learns a similarity metric for face matching—Reduces the intra-class variations
A real-time cnn-based lightweight mobile masked face recognition system	MobileNetV2 with SSD for mask detection and ArcFace for face recognition	- Prone to false positives or negatives in mask detection—May not work well on low-resolution images	- Lightweight and efficient for mobile devices—Achieves high accuracy on masked faces
[Face recognition in the scene of wearing a mask]	CNN with mask removal module and attention mechanism	- Relies on the quality of mask removal—May introduce artifacts or distortions in the reconstructed faces	- Recovers the occluded facial regions—Focuses on the important facial features
[Face mask detection using YOLOv3 and faster R-CNN models: COVID-19 environment]	YOLOv3 and Faster R-CNN for mask detection	- YOLOv3 may have lower precision than Faster R-CNN—Faster R-CNN may have lower speed than YOLOv3	- Both models have high recall and accuracy for mask detection—Both models can handle multiple faces in an image
[Masked face detection algorithm in the dense crowd based on federated learning]	Federated learning with CNN for mask detection	- Requires secure and reliable communication among devices—May have privacy or security risks in data sharing	- Reduces the data transmission cost and latency—Preserves the data privacy and sovereignty
[Face mask detection using transfer learning of inceptionv3]	InceptionV3 with transfer learning from ImageNet for mask detection	- Needs fine-tuning for different data sets or domains—May overfit or underfit the data depending on the parameters	- Exploits the pre-trained weights from ImageNet—Captures complex features with multiple filters
[A hybrid deep transfer learning model with machine learning methods for face mask detection in the era of the COVID-19 pandemic]	VGG16 with transfer learning and SVM or KNN for mask detection	- VGG16 is a large and complex model that may be slow or memory-intensive—SVM or KNN may not be optimal for high-dimensional features	- Leverages the transfer learning from VGG16—Combines deep learning and machine learning methods
[Face mask detection by using optimistic convolutional neural network]	CNN with dropout and batch normalization for mask detection	- Dropout may reduce the effective capacity of the model—Batch normalization may introduce extra computation or complexity	- Optimizes the model performance and generalization—Prevents overfitting and accelerates convergence
[Prototype for integration of face mask detection and person identification model–COVID-19]	CNN with MobileNetV2 for mask detection and ResNet50 for face recognition	- MobileNetV2 may have lower accuracy than other models—ResNet50 may have lower speed than other models	- Integrates mask detection and face recognition in one system—Uses lightweight and powerful models for both tasks
[Evaluating the masked and unmasked face with LeNet algorithm]	LeNet with data augmentation for face recognition	- LeNet is a simple and shallow model that may not capture complex features—Data augmentation may not be sufficient to handle large variations	- Suitable for small and low-resolution images—Improves the data diversity and robustness
[A simple mask detection model based on a multi-layer perception neural network]	MLP with ReLU activation and softmax output for mask detection	- MLP is a basic and linear model that may not learn non-linear features—MLP may have high bias or variance depending on the number of layers or neurons	- Easy to implement and train—Has low computational cost and complexity
[Masked face recognition algorithm for a contactless distribution cabinet]	CNN with mask segmentation and feature fusion for face recognition	- Requires accurate segmentation of masks from faces—May have difficulty in fusing features from different sources	- Segments the masks from the faces to reduce occlusion—Fuses the features from masked and unmasked regions
[Control the COVID-19 pandemic: Face mask detection using transfer learning]	CNN with transfer learning from VGG19 for mask detection	- VGG19 is a large and complex model that may be slow or memory-intensive—Transfer learning may not adapt well to new data sets or domains	- Exploits the pre-trained weights from VGG19—Achieves high accuracy on mask detection

**Table 2:** Performance of our proposed methods compared to the other methods in terms of accuracy in masked face recognition.

Reference	Data Set	Method	Recognition Type	Val. Acc.
Ejaz et al. [45]	ORL Face [47]	PCA	Masked	72%
Sikha et al. [48]	Masked Georgia tech face data set	A hybrid VGG16-Random Fourier deep learning model	Masked	85.9%
Li et al. [49]	Webface, AR, Yela B, LFW	Face cropping and CBAM	Masked	92.6%
Ding et al. [11]	CASIA-WebFace [50]	Latent Part Detection	Masked	94%
Wu [24]	RMFRD and SMFRD databases of Wuhan University	Attention mechanism	Masked	95.3%
Rodriguez et al. [46]	N/A	Mixture of Gaussian	Masked	95.4%
Lane et al. [51]	Celeb-A, LFW, AR	De-occlusion distillation	Masked	95.4%
Proposed method	LFW-SMFD [44] and real data set	Deep learning, PCA, and KNN	Masked	97%

**Table 3:** Performance of our proposed methods compared to the other methods in terms of accuracy in unmasked face image.

Reference	Data Set	Method	Recognition Type	Val. Acc.
Ejaz et al. [45]	ORL Face [47]	PCA	Unmasked	95%
Ding et al. [11]	CASIA-WebFace [50]	Latent Part Detection	Unmasked	97%
DeepFace [52]	LFW	deep learning	Unmasked	97.3%
Wu [24]	RMFRD and SMFRD databases of Wuhan University	Attention mechanism	Unmasked	98.3%
Proposed method	LFW-SMFD [44] and real data set	Hybrid method using deep learning, PCA, and KNN	Unmasked	98.4%

**Table 4:** PSO parameters.

Parameter	Value
Swarm size	50
Inertia weight	1
Personal learning coefficient	1.5
Global learning coefficient	2.0

**Table 5:** Comparative analysis of accuracy of proposed method with existing benchmark algorithms in case of unmasked data set.

Type of Features	KNN Acc.	PSO-KNN Acc.
Actual features	89%	93%
GA	94%	96%
PSO	95%	98%

## Data Availability

The data that support the findings of this study are available in https://www.kaggle.com/datasets/muhammeddalkran/lfw-simulated-masked-face-dataset, accessed on 26 June 2023.
